# Long-term outcomes after thoracoscopic versus open surgery for congenital esophageal atresia: propensity-score overlap weighting analysis

**DOI:** 10.1007/s00383-025-06135-2

**Published:** 2025-07-22

**Authors:** Yoshitsugu Yanagida, Shotaro Aso, Michimasa Fujiogi, Kaori Morita, Mai Kutsukake, Naohiro Takamoto, Kiyohide Fushimi, Jun Fujishiro, Hideo Yasunaga

**Affiliations:** 1 Department of Pediatric Surgery, Graduate School of Medicine, The University of Tokyo, 7-3-1 Hongo, Bunkyo-ku, Tokyo Japan; 2 Department of Clinical Epidemiology and Health Economics, School of Public Health, The University of Tokyo, 7-3-1 Hongo, Bunkyo-ku, Tokyo Japan; 3 Department of Health Services Research, Graduate School of Medicine, The University of Tokyo, 7-3-1 Hongo, Bunkyo-ku, Tokyo Japan; 4 Division of Surgery, Department of Surgical Specialties, National Center for Child Health and Development, 2-10-1 Okura, Setagaya-ku, Tokyo Japan; 5 Department of Health Informatics and Policy, Graduate School of Medicine, Institute of Science Tokyo, Tokyo, Japan

**Keywords:** Congenital esophageal atresia, Thoracoscopic surgery, Open surgery, Neonatal surgery, Long-term outcome

## Abstract

**Purpose:**

Congenital esophageal atresia requires neonatal surgery. Although short- and medium-term outcomes of thoracoscopic and open surgery have been investigated, long-term outcomes remain unclear. This study aimed to compare the long-term outcomes of these approaches using a Japanese national inpatient database.

**Methods:**

We identified neonates who underwent open or thoracoscopic surgery for congenital esophageal atresia between April 2016 and March 2022. Patients with prior palliative surgery were excluded. Propensity score overlap weighting analyses were used to compare the outcomes between the groups. The primary outcome was the long-term (1 year after definitive surgery) anastomotic strictures. Secondary outcomes included long- and medium-term (within 30 days to 1 year after definitive surgery) gastroesophageal reflux and medium-term anastomotic strictures.

**Results:**

Among 395 patients, 67 underwent thoracoscopic surgery and 328 underwent open surgery.　Propensity score overlap weighting analyses revealed no significant differences in long-term anastomotic stricture (5.8% vs. 8.7%; risk difference (RD), − 2.9%; 95% confidence interval (CI), − 10.9 to 5.1), long-term gastroesophageal reflux (2.9% vs. 3.0%; RD, -0.1%; 95% CI − 7.1 to 4.9), medium-term anastomotic stricture (29.4% vs. 18.8%; RD, 10.6%; 95% CI − 2.5 to 23.7), or medium-term gastroesophageal reflux (4.9% vs. 6.0%; RD, − 1.1; 95% CI −7 .1 to 4.9).

**Conclusions:**

Long-term outcomes did not differ significantly between thoracoscopic and open surgery for congenital esophageal atresia.

## Introduction

Congenital esophageal atresia is reported to occur in approximately 1 in 2500–4000 live births [1] and requires surgical intervention during the neonatal period. Since the first report of thoracoscopic surgery for congenital esophageal atresia in 2000 [2], the number of institutions that perform thoracoscopic surgery for this condition has gradually increased.

Several observational studies and meta-analyses which compared surgical outcomes between thoracoscopic and open surgery for esophageal atresia have reported a similar proportion of postoperative complications, such as anastomotic leakage, anastomotic stricture, and gastroesophageal reflux requiring fundoplication [3–7]. However, these studies evaluated the surgical outcomes without adjusting for patient background.

A previous study using propensity score overlap weighting analysis revealed no significant difference in complications between thoracoscopic and open surgery for esophageal atresia. However, this study did not evaluate long-term complications [8]. Similarly, another study that used propensity score-matching analysis evaluated only short-term outcomes (within 30 days after definitive surgery) [9]. Thus, long-term outcomes adjusted for patient backgrounds remain uninvestigated.

We aimed to compare the long-term surgical outcomes of thoracoscopic and open surgery for congenital esophageal atresia while accounting for differences in patient background.

## Materials and methods

### Data source

This nationwide retrospective cohort study used data from the Diagnosis Procedure Combination database of Japan. More than 1200 hospitals voluntarily contributed to the database, which includes data on approximately 8,000,000 inpatients annually. All 82 academic hospitals in Japan participated in the database [10]. Approximately 90% of hospitals with neonatal intensive care units were included in this database [10, 11]. The database includes the following data: unique hospital identifiers, patient age, body weight, body height, sex, diagnoses, comorbidities at admission, and complications after admission recorded as text data in Japanese and the International Classification of Diseases, Tenth Revision (ICD-10) codes [12]. Previous studies have shown that the validity of diagnoses, procedural records, and operative information in the databases is high [13, 14].

This study was conducted in accordance with the STROCSS criteria [15]. The requirement for informed consent was waived because the patient data were anonymized. This study was approved by our institutional review board (Approval number: 3501-(5), May 19, 2021).

### Patient selection

In Japan, thoracoscopic surgery for congenital esophageal atresia was covered by public health insurance in April 2016. We identified patients who underwent definitive surgery (thoracoscopic or open surgery) for congenital esophageal atresia during their initial hospitalization between April 2016 and March 2022. We excluded patients who (i) underwent palliative surgery (gastrostomy, exploratory thoracotomy, or laparotomy) prior to definitive surgery, and (ii) underwent definitive surgery after 1 week of age without palliative surgeries. These patients were excluded because they were considered to have an atypical clinical course or more complicated conditions, which could introduce bias in comparing surgical outcomes. Additionally, only a small number of patients aged 7–14 days underwent definitive surgery without prior palliative procedure. Including them would have had minimal impact on group sizes (only five additional patients in the open surgery group and none in the thoracoscopic group).We categorized eligible patients into thoracoscopic and open surgery groups. We also recorded all rehospitalizations for each eligible patient for up to 6 years after initial discharge. 

### Outcomes and covariates

The primary outcome was long-term anastomotic stricture. The secondary outcomes were long-term gastroesophageal reflux, medium-term anastomotic stricture, and gastroesophageal reflux, anastomotic leakage, short-term anastomotic stricture, duration of anesthesia, reoperation, in-hospital mortality, duration of postoperative mechanical ventilation, length of postoperative neonatal intensive care unit (NICU) stay, length of postoperative hospital stay, and total cost of hospitalization. We defined anastomotic stricture as the need for operative dilation using any type of dilating method (including balloon dilation and bougienage) after definitive surgery, based on a previous study [16]. We defined gastroesophageal reflux as the requirement for fundoplication after definitive surgery. “Short-term” was defined as requiring initial dilation within 30 days after definitive surgery, as in a previous study [10]. “Medium-term” and “long-term” were defined as requiring an initial procedure (dilation or fundoplication) within 30 days to 1 year and 1 year or later after definitive surgery, respectively. Anastomotic strictures and gastroesophageal reflux are likely to occur within the first year after definitive surgery for congenital esophageal atresia [17, 18]. We defined anastomotic leakage as both a recorded diagnosis of anastomotic leak after admission and the need for continuous suction drainage for 3 weeks after definitive surgery based on a previous study [16]. We defined the currency exchange rate as 140 Japanese yen per 1 US dollar.

The covariates included sex, age, birth weight, gestational age (in week), and age at surgery (in days), congenital heart disease (ICD-10 codes: Q20–26), congenital malformations of the respiratory system (ICD-10 codes: Q30–34), congenital malformations of the digestive system (ICD-10 codes: Q38–45), congenital malformation and deformation of the musculoskeletal system disease (ICD-10 codes: Q65–79), trisomy 21 (ICD-10 codes: Q90), trisomy 18 (ICD-10 codes: Q91.0–3), and trisomy 13 (ICD-10 codes: Q91.4–7), birth asphyxia, use of vasopressor (at the day of surgery), and transfusion (on the day of surgery), hospital volume, and fiscal year.

Body weight was categorized into the following three groups based on the World Health Organization definition of low birth weight [19]: < 1500 g, 1500–2500 g, and > 2500 g. Gestational age was categorized into two groups: < 37 weeks and ≥ 37 weeks. Hospital volume was defined as the average annual number of definitive operations for esophageal atresia performed at each hospital over 6 years and was categorized into tertile: 0 > case and < 1 case, ≥ 1 case and < 2 cases, and ≥ 2 cases. Patent ductus arteriosus was defined based on ICD-10 codes and operations for congenital heart disease [20]. We defined mild birth asphyxia as neonatal resuscitation for first-degree birth asphyxia (Apgar scores of 4–6) and severe birth asphyxia as neonatal resuscitation for second-degree birth asphyxia (Apgar scores of 0–3) [21].

### Statistical analysis

We used Fisher’s exact test and the chi-squared test to compare the proportions of categorical variables, and the Mann–Whitney U test to compare the medians of continuous variables.

To compare the perioperative outcomes between the groups, we conducted a propensity score overlap weighting analysis [22, 23]. First, we calculated the propensity scores using a logistic regression model with patients undergoing thoracoscopic surgery as the dependent variable. The independent variables included sex, age at surgery, gestational age, birth weight, congenital heart disease, trisomy 18, vasopressor use, blood transfusion use, mild and severe birth asphyxia, congenital malformation (respiratory, digestive, and musculoskeletal systems), hospital volume, and fiscal year. Patients in the thoracoscopic surgery group were weighted according to the probability of not undergoing thoracoscopic surgery (1-propensity score), and those in the open surgery group were weighted according to the probability of undergoing thoracoscopic surgery (propensity score). Propensity score overlap weighting analysis attempts to mimic the important attributes of randomized clinical trials, such as the target population, covariate balance, and precision, which are potential limitations of conventional propensity score methods of inverse probability of treatment weighting and matching [24]. We calculated standardized differences to examine the balance of baseline characteristics between the groups before and after propensity score overlap weighting. A standardized difference of less than 0.1 was considered a negligible imbalance between the two groups [25].

We considered a significant level as P < 0.05 for all statistical tests, with all reported P values being two-sided. Statistical analyses were conducted using Stata/SE 18.0 (Stata Corp., College Station, TX, USA).

## Results

**Fig. 1:**
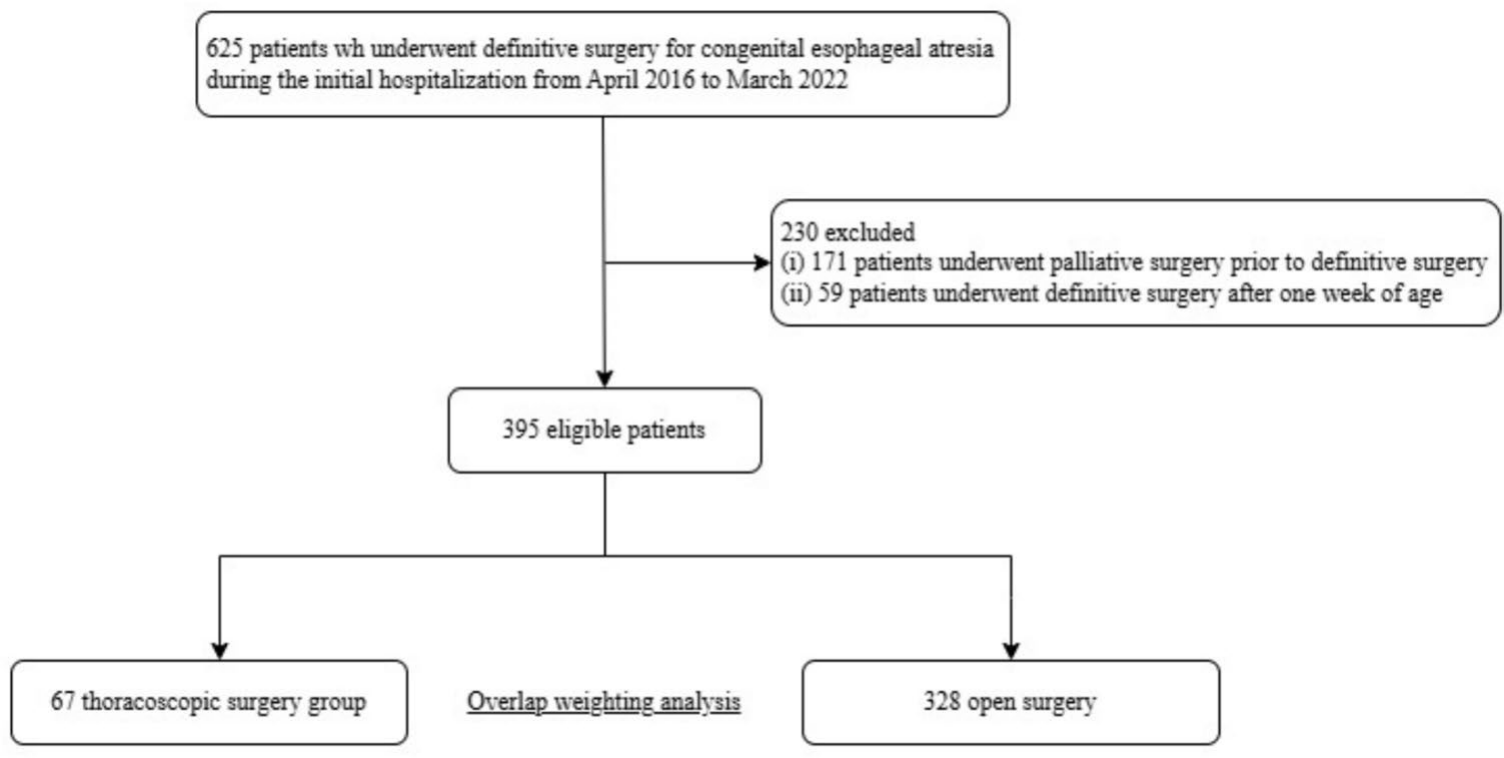
Flow diagram. Flow diagram for patients who underwent thoracoscopic or open surgery for esophageal atresia

We identified 625 patients who underwent definitive surgery for congenital esophageal atresia during their initial hospitalization between April 2016 and March 2022. We excluded 171 patients who underwent palliative surgery before definitive surgery and 59 patients who underwent definitive surgery after 1 week of age. Of the eligible 395 patients, 67 underwent thoracoscopic surgery and 328 underwent open surgery (Fig. 1).

The baseline characteristics of the patients before and after propensity score overlap weighting are presented in Table 1. Before overlap weighing, patients in the thoracoscopic surgery group had higher birth weight, underwent surgery at a higher-volume hospital, and were less likely to have congenital heart disease compared to those in the open surgery group.

**Table 1:** Baseline characteristics of all patients and propensity score-weighted patients

	All patients					Propensity-score overlap weighted patients		
	Thoracoscopic surgery		Open surgery		Standardized difference	Thoracoscopic surgery	Open surgery	Standardized difference
	n = 67		n = 328					
Sex, male, n(%)	36	(53.7)	174	(53.0)	0.01	(52.6)	(52.6)	< 0.01
Age at surgery, n(%)								
0 day	7	(10.4)	82	(25.0)	0.51	(13.3)	(13.3)	< 0.01
1 day	35	(52.2)	140	(42.7)		(57.8)	(57.8)	
2 days	15	(22.4)	61	(18.6)		(15.6)	(15.6)	
3 days	6	(9.0)	22	(6.7)		(9.2)	(9.2)	
4 days	1	(1.5)	11	(3.4)		(2.3)	(2.3)	
5 days	1	(1.5)	6	(1.8)		(1.7)	(1.7)	
6 days	0	0.0	5	(1.5)		0.0	0.0	
7 days	2	(3.0)	1	(0.3)		0.0	0.0	
Preterm gestational age (< 37 weeks), n(%)	17	(25.4)	76	(23.2)	0.05	(27.5)	(27.5)	< 0.01
Birth weight, n(%)								
< 1500	0	0.0	6	(1.8)	0.29	0.0	0.0	< 0.01
1500–2500	26	(38.8)	147	(44.8)		(44.4)	(44.4)	
> 2500	37	(55.2)	155	(47.3)		(55.6)	(55.6)	
missing	4	(6.0)	20	(6.1)		0.0	0.0	
Birth asphyxia, n(%)	4	(6.0)	33	(10.1)	0.15	(7.0)	(7.0)	< 0.01
Severe birth asphyxia, n(%)	5	(7.5)	21	(6.4)	0.04	(8.1)	(8.1)	< 0.01
Congenital malformation, n(%)								
Heart disease	5	(7.5)	61	(18.6)	0.34	(9.9)	(9.9)	< 0.01
Digestive system	12	(17.9)	67	(20.4)	0.06	(19.8)	(19.8)	< 0.01
Respiratory system	3	(4.5)	24	(7.3)	0.12	(5.8)	(5.8)	< 0.01
Musculoskeletal system	6	(9.0)	23	(7.0)	0.07	(8.8)	(8.8)	< 0.01
Trisomy 21	0	0.0	0	0.0	0.00	0.0	0.0	< 0.01
Trisomy 18	0	0.0	7	(2.1)	0.21	0.0	0.0	< 0.01
Trisomy 13	0	0.0	0	0.0	0.00	0.0	0.0	< 0.01
Vasopressor use, n(%)	15	(22.4)	78	(23.8)	0.03	(25.0)	(25.0)	< 0.01
Transfusion, n(%)	3	(4.5)	26	(7.9)	0.14	(5.8)	(5.8)	< 0.01
Annual hospital volume for congenital esophageal atresia, n(%)								
0 > , < 1 case	6	(10.2)	111	(36.2)	0.57	(15.8)	(15.8)	< 0.01
≥ 1, < 2 cases	23	(39.0)	116	(37.8)		(33.3)	(33.3)	
≥ 2 cases	30	(50.8)	80	(26.1)		(50.9)	(50.9)	
Fiscal year, n(%)								
2016	11	(16.4)	52	(15.9)	0.44	(15.8)	(15.8)	< 0.01
2017	8	(11.9)	81	(24.7)		(16.4)	(16.4)	
2018	10	(14.9)	68	(20.7)		(15.8)	(15.8)	
2019	12	(17.9)	47	(14.3)		(15.8)	(15.8)	
2020	13	(19.4)	38	(11.6)		(18.7)	(18.7)	
2021	13	(19.4)	42	(12.8)		(17.5)	(17.5)	

Data are presented as mean (SD) for continuous variables and n (%) for categorical variables

The crude outcomes for all patients are shown in Table 2. No significant difference was observed in the proportions of long-term anastomotic stricture (4.5% vs. 8.5%, P = 0.26), long-term gastroesophageal reflux (3.0% vs 2.4%, P = 0.80), medium-term anastomotic stricture (29.9% vs 23.2%, P = 0.25), medium-term gastroesophageal reflux (7.5% vs.4.6%, P = 0.33), the average duration of anesthesia (249.5 (95% confidential interval (CI) 219.4–279.6) min vs. 277.8 (265.4–290.2) min, P = 0.07), anastomotic leakage (7.5% vs. 6.7%, P = 0.82), short-term anastomotic stricture (3.0% vs. 3.1%, P = 0.98), reoperation (4.5% vs. 2.7%, P = 0.45), in-hospital mortality (1.5% vs. 3.7%, P = 0.37), the average duration of postoperative mechanical ventilation (54.5 (28.4–80.5) days vs. 44.4 (29.8–58.9) days, P = 0.56), the average length of postoperative NICU stay (26.5 (22.2–30.7) days vs. 24.4 (23.3–25.5) days, P = 0.18), the average length of postoperative hospital stay (83.6 (59.1–108.1) days vs. 86.8 (70.6–102.9) days, P = 0.87), and the average total cost of hospitalization (70,053 (60,163–79,943) US dollars vs. 67,831 (61,553–74,110) US dollars, P = 0.76). No short-term gastroesophageal reflux was observed in either the thoracoscopic or open surgery groups.

**Table 2:** Crude outcomes of all patients

Outcomes	Thoracoscopic surgery		Open surgery		P-value
	n = 67		n = 328		
Long-term					
AS, n(%)	3	(4.5)	28	(8.5)	0.26
GER, n(%)	2	(3.0)	8	(2.4)	0.80
Middle-term					
AS, n(%)	20	(29.9)	76	(23.2)	0.25
GER, n(%)	5	(7.5)	15	(4.6)	0.33
Perioperative					
Duration of anesthesia, min(SD)	249.5	(123.3)	277.8	(114.4)	0.07
Anastomotic leakage, n(%)	5	(7.5)	22	(6.7)	0.82
Short-term AS, n(%)	2	(3.0)	10	(3.1)	0.98
Reoperation, n(%)	3	(4.5)	9	(2.7)	0.45
In-hospital mortality, n(%)	1	(1.5)	12	(3.7)	0.37
Postoperative ventilatory duration, days(SD)	54.5	(105.9)	44.4	(133.1)	0.56
Postoperative NICU stay, days (SD)	26.5	(17.3)	24.4	(10.0)	0.18
Postoperative hospital stay, days (SD)	83.6	(100.5)	86.8	(148.5)	0.87
Total cost of hospitalization, USD (SD)	70,053	(40,547)	67,831	(57,798)	0.76

Data are presented as mean (SD) for continuous measures, and n (%) for categorical measures

*AS* anastomotic stricture, *GER* gastroesophageal reflux, *NICU* neonatal intensive care unit, *USD* US dollars, *SD* standard deviation

In the overlap weight analysis, all baseline characteristics were well-balanced (Table 1). No significant difference was observed between the groups in long-term anastomotic stricture (5.8% vs. 8.7%; risk difference, (RD) −2.9%; 95% CI −10.9 to 5.1), long-term gastroesophageal reflux (RD, − 0.1%; 95% CI − 5.3 to 5.1), medium-term anastomotic stricture (RD, 10.6%, 95% CI − 2.5 to 23.7), medium-term gastroesophageal reflux (RD, − 1.1, 95% CI − 7.1 to 4.9), mean duration of anesthesia (difference, -19.9 min; 95% CI − 55.7 to 16.0), anastomotic leakage (RD, 0.6%; 95% CI − 8.0 to 9.1), short-term anastomotic stricture (RD, −0.4, 95% CI −6.0 to 5.2), reoperation (RD, 3.8%; 95% CI −2.0 to 9.6), in-hospital mortality (RD, 1.2%; 95% CI − 1.7 to 4.1), mean duration of postoperative mechanical ventilation (difference, 14.7 days; 95% CI − 20.8 to 50.3), mean length of postoperative NICU stay (difference, 3.5 days; 95% CI −0.7 to 7.7), mean length of postoperative hospital stay (difference, − 2.6 days, 95% CI − 38.3 to 33.2), or mean total cost of hospitalization (difference, 3495 US dollars; 95% CI − 10,613 to 17,603) (Table 3).

**Table 3:** Outcome analysis after propensity score overlap weighting

Outcomes	Thoracoscopic surgery		Open surgery		Risk difference	(95% CI)	P value
Long-term							
AS, %	5.8		8.7		− 2.9	(− 10.9–5.1)	0.48
GER, %	2.9		3.0		− 0.1	(− 5.3–5.1)	0.97
Middle-term							
AS, %	29.4		18.8		10.6	(− 2.5–23.7)	0.11
GER, %	4.9		6.0		− 1.1	(− 7.1–4.9)	0.71
Perioperative							
Duration of anesthesia, min(SD)	254.9	(120.1)	274.7	(107.9)	− 19.9	(− 55.7–16.0)	0.28
Anastomotic leakage, %	8.2		7.7		0.6	(− 8.0–9.1)	0.90
Short-term AS, %	2.9		3.3		− 0.4	(− 6.0–5.2)	0.88
Reoperation, %	5.0		1.2		3.8	(− 2.0–9.6)	0.20
In-hospital mortality, %	1.5		0.3		1.2	(− 1.7–4.1)	0.43
Postoperative ventilatory duration, days (SD)	58.3	(108.8)	43.5	(153.5)	14.7	(− 20.8–50.3)	0.42
Postoperative NICU stay, days(SD)	26.7	(16.4)	23.1	(9.2)	3.5	(− 0.7–7.7)	0.10
Postoperative hospital stay, days(SD)	86.4	(103.0)	89.0	(170.8)	− 2.6	(− 38.3–33.2)	0.89
Total cost of hospitalization, USD (SD)	70,955	(41,224)	67,460	(65,459)	3495	(− 10,613–17,603)	0.63

Data are presented as mean (SD) for continuous measures and % for categorical measures

*AS* anastomotic stricture, *GER* gastroesophageal reflux, *NICU* neonatal intensive care unit, *USD* US dollars, *SD* standard deviation

## Discussion

In this study, we compared the long-term outcomes of thoracoscopic and open surgery for congenital esophageal atresia using a national inpatient database in Japan. Propensity score overlap weighting analyses revealed no significant differences in long-term outcomes between the two groups. This is the first report to compare the proportions of long-term anastomotic strictures and gastroesophageal reflux between thoracoscopic and open surgery, with adjustment for patient characteristics.

The current studies found no significant difference in the proportion of short- and medium-term outcomes, such as anastomotic leakage and anastomotic stricture within 1 year after definitive surgery. These findings are consistent with previous research [3–8]. The exclusion of patients who underwent palliative procedures before definitive surgery may have contributed to these findings by limiting the inclusion of cases with a long-gap esophageal atresia, which is a known risk factor for anastomotic leakage and strictures [26, 27].

Our findings revealed no significant differences in long-term anastomotic strictures or gastroesophageal reflux between groups. One possible reason for this is that we excluded patients who underwent palliative procedures prior to definitive surgery owing to a long gap. A long gap is strongly associated with anastomotic stricture and gastroesophageal reflux. Patients requiring staged repair are more likely to develop these complications because a long gap increases the anastomotic tension associated with an anastomotic leak and subsequent anastomotic strictures [17, 28]. Higher tension at the anastomotic site can retract the lower esophagus upward and induce anatomical changes at the gastroesophageal junction [29]. Our findings revealed no significant difference in the proportion of anastomotic strictures or gastroesophageal reflux between groups. These findings indicate that thoracoscopic and open surgeries could have similar impacts on the tension at the anastomotic site related to anastomotic stricture, and anatomical and functional changes at the gastroesophageal junction associated with gastroesophageal reflux.

Although no significant differences in long-term anastomotic stricture and gastroesophageal reflux were observed between thoracoscopic and open surgery for esophageal atresia in this study, a comparison of outcomes between thoracoscopic and open surgery for esophageal atresia may be premature. Thoracoscopic surgery for esophageal atresia has not been widely performed, and the expertise in thoracoscopic surgery may be insufficient due to the rarity of esophageal atresia. Thoracoscopic surgery for esophageal atresia was included under public health insurance in Japan in 2016, and our study period spanned from 2016 to 2022. A previous study reported a steep learning curve for thoracoscopic surgery (approximately 10–20 cases) for esophageal atresia [30]. Moreover, the level of expertise of surgeon performing thoracoscopic surgery may differ from those performing open surgery. Most surgeons who perform thoracoscopic surgery are certified expert surgeons, whereas those who perform open surgery are likely to be surgical trainees [31]. In the future, as thoracoscopic surgery for congenital esophageal atresia is more widely adopted, further studies adjusting for both patient backgrounds and surgeon levels will be required. 

However, our study had certain limitations. First, we could not detect the type of congenital esophageal atresia (Gross classification) or gap length in the database. To align patient backgrounds, we excluded patients who underwent palliative surgery (such as gastrostomy) before definitive surgery and those who underwent definitive surgery after 1 week of age. Second, we were unable to follow up with patients across different hospitals in this database. Therefore, we may have underestimated the proportion of patients with anastomotic strictures and gastroesophageal reflux. Third, the Diagnosis Procedure Combination database did not include the results of tests for anastomotic stricture and gastroesophageal reflux, such as esophagogastrography, endoscopy, and pH-monitoring tests. Thus, we were unable to evaluate the severity of anastomotic stricture and gastroesophageal reflux. In addition, we could not define the anastomotic leakage owing to the lack of data on esophagograms and patients’ symptoms. Fourth, we could not detect conversions from thoracoscopic to open surgery. The proportion of conversions varied among previous studies (3.2–53%) [3–7]. Therefore, we may have underestimated the effects of thoracoscopic surgery. Fifth, the database did not provide information on other important outcomes, such as thoracic deformity, including scoliosis, which has been reported as a long-term complication of open thoracotomy [32, 33].

## Conclusion

No significant differences were observed in the long-term outcomes between thoracoscopic surgery and open surgery for congenital esophageal atresia. Using a national inpatient database in Japan and applying propensity score overlap weighting analyses, we provided the first adjusted comparison of these long-term outcomes. Our findings suggest that both surgical approaches may have similar impacts on anastomotic tension and anatomical changes at the gastroesophageal junction.

## Data Availability

No datasets were generated or analysed during the current study.
